# Associations between plasma biomarkers and brain amyloid and tau pathologies in a population‐based cohort without evidence of cognitive impairment

**DOI:** 10.1002/alz.092544

**Published:** 2025-01-09

**Authors:** Menayit Tamrat Dresse, Pamela C.L. Ferreira, Bruna Bellaver, Xuemei Zeng, Anuradha Sehrawat, Yijun Chen, Guilherme Povala, Tara K Lafferty, Victor L Villemagne, M. Ilyas Kamboh, Ann D Cohen, William E Klunk, Tharick A. Pascoal, Mary Ganguli, C. Elizabeth Shaaban, Beth E. Snitz, Thomas K Karikari

**Affiliations:** ^1^ University of Pittsburgh, Pittsburgh, PA USA; ^2^ University of Pittsburgh Alzheimer's Disease Research Center (ADRC), Pittsburgh, PA USA; ^3^ University of Gothenburg, Mölndal Sweden

## Abstract

**Background:**

Plasma biomarkers have been widely evaluated as surrogate markers for brain Alzheimer’s disease (AD) pathology. However, studies in diverse populations with lower socioeconomic status (SES) are limited. We evaluated associations between different plasma biomarkers and brain amyloid beta (Aβ) and tau positron emission tomography (PET) in a longitudinal cohort of cognitively normal participants from a medically‐ and economically‐underserved Rust Belt region of Western Pennsylvania, USA.

**Method:**

We measured plasma Aβ42/40, p‐tau181, p‐tau231, p‐tau217, NfL, and GFAP at baseline (n=111) and in a subset (n=65) at two‐year follow‐up in the MYHAT‐NI cohort. Aβ PET and tau PET were assessed using [^11^C] PiB and [^18^F] AV‐1451, respectively, and level of functioning with Clinical Dementia Rating (CDR). We included only cognitively normal participants (CDR = 0).

**Result:**

The baseline median age was 76.7 years; 60 (54.1%) were females, and 18 (16.2%) were APOE ε4 carriers. The follow‐up cohort had a median age of 77.7 years, with 12 (18.5%) APOE ε4 carriers and 33 females (50.8%). All plasma biomarkers showed stronger associations with Aβ PET standard uptake value ratio (SUVR) in the Aβ‐positive vs. negative participants. However, only the associations for plasma p‐tau217 (rho=0.74, P<0.001), p‐tau231 (rho=0.48, P=0.02) and Aβ42/40 (rho= ‐0.43, P=0.04) in the Aβ PET‐positive group were significant. Voxel‐wise association models of Aβ PET accounting for age, sex, and CDR showed the strongest associations in the following decreasing order for plasma p‐tau217>Aβ42/40>GFAP>p‐tau181. No significant correlation with tau PET SUVR was observed for the plasma biomarkers.

**Conclusion:**

Plasma p‐tau217 showed the strongest association with neuroimaging measures of brain Aβ in this cognitively normal group of older adults with lower SES. These results suggest that plasma p‐tau217 might be important to screen for evidence of brain amyloidosis in cognitively normal individuals. Future work will investigate the effects of specific comorbidities and demographic factors on the outcomes and if these change over the course of the two‐year follow‐up period.

